# Petahertz non-linear current in a centrosymmetric organic superconductor

**DOI:** 10.1038/s41467-020-17776-3

**Published:** 2020-08-18

**Authors:** Y. Kawakami, T. Amano, H. Ohashi, H. Itoh, Y. Nakamura, H. Kishida, T. Sasaki, G. Kawaguchi, H. M. Yamamoto, K. Yamamoto, S. Ishihara, K. Yonemitsu, S. Iwai

**Affiliations:** 1 Department of Physics, Tohoku University, Sendai, 980-8578 Japan; 2 Department of Applied Physics, Nagoya University, Nagoya, 464-8603 Japan; 3 Institute for Materials Research, Tohoku University, Sendai, 980-8577 Japan; 4 Institute for Molecular Science, Okazaki, 444-8585 Japan; 5 Department of Applied Physics, Okayama University of Science, Okayama, 700-0005 Japan; 6 Department of Physics, Chuo University, Tokyo, 112-8551 Japan

**Keywords:** Nonlinear optics, Organic molecules in materials science, High-harmonic generation

## Abstract

Charge acceleration during an intense light field application to solids attracts much attention as elementary processes in high-harmonic generation and photoelectron emission. For manipulating such attosecond dynamics of charge, carrier-envelope-phase (CEP: relative phase between carrier oscillation of light field and its envelope function) control has been employed in insulators, nanometal and graphene. In superconducting materials, collective control of charge motion is expected because of its strongly coherent nature of quasi-particles. Here we report that, in a layered organic superconductor, a non-linear petahertz current driven by a single-cycle 6 femtosecond near infrared field shows up as second harmonic generation (SHG), which is in contrast to the common belief that even harmonics are forbidden in the centrosymmetric system. The SHG represents a CEP sensitive nature and an enhancement near the superconducting temperature. The result and its quantum many-body analysis indicate that a polarized current is induced by non-linear acceleration of charge, which is amplified by superconducting fluctuations. This will lead to petahertz functions of superconductors and of strongly correlated systems.

## Abstract (web summary)

Here the authors show second harmonic generation (SHG) from a centrosymmetric organic superconductor κ-(BEDT-TTF)_2_Cu[N(CN)_2_]Br. They find unusual temperature dependence and CEP-sensitive nature of the SHG which are explained in terms of nonlinear current.

## Introduction

An electromagnetic oscillation of light cannot directly drive a polarized current because of its symmetric nature on the time axis (i.e., the time average of the oscillation is zero). However, recent developments of ultrashort laser technologies enable us to control the direction of charge motion by carrier-envelope phase (CEP) control of a strong light field^1–12^. Considering non-linear light–matter interactions during an ultrashort pulse, we can expect petahertz control of the polarized current in superconducting materials.

**Fig. 1:**
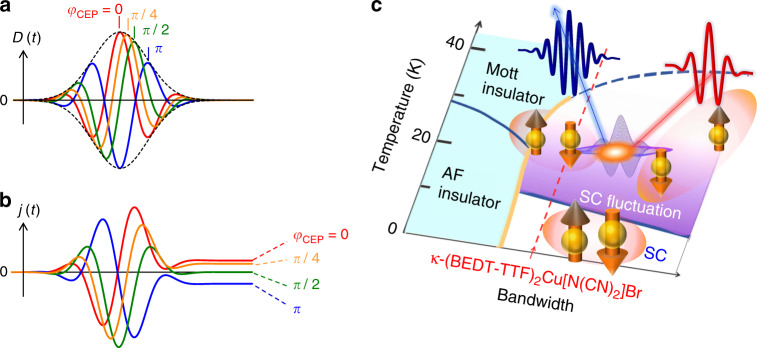
CEP control of non-linear current and phase diagram of κ-type BEDT–TTF salts. **a** Non-linear electric displacement *D*(*t*) induced by electric field $E\left( t \right) = E_0\left( t \right){\mathrm{sin}}\left( {\omega t - \varphi _{{\mathrm{CEP}}}} \right)$, where $\varphi _{{\mathrm{CEP}}}$ = 0 (red), 1/4*π* (orange), 1/2*π* (green), *π* (blue) (*E*_0_(*t*): envelope of a single-cycle pulse) (we assume $\left. {D\left( t \right) \propto {\mathrm{sin}}\left( {\omega t - \varphi _{{\mathrm{CEP}}}} \right)} \right]$. **b** Non-linear light-induced current ${j}\left( t \right) \propto \mathop {\smallint }\limits_0^t D\left( \tau \right){\mathrm{{d}}}\tau$. **c** Temperature-*t*/*U*_dimer_(band width) phase diagram of κ-(BEDT–TTF)_2_X, which is extracted based on controlling the chemical pressure (=*t*/*U*_dimer_).

In the non-linear regime (where a non-linear electric displacement along the polarization axis *D*(*t*) is induced by an electric field), a net current given by $j\left( t \right) \propto \mathop {\smallint }\limits_0^t D\left( \tau \right){\mathrm{{d}}}\tau$ does not necessarily vanish for a few-cycle or single-cycle field. Interestingly, such a non-linear current can be modulated by the CEP as shown in Fig. 1b, if the CEP of $E\left( t \right) = E_0\left( t \right){\mathrm{sin}}\left( {\omega t - \varphi _{{\mathrm{CEP}}}} \right)$ (*E*_0_ (*t*): envelope function of a single-cycle pulse, $\varphi _{{\mathrm{CEP}}}$: CEP) is preserved in $D\left( t \right) \propto {\mathrm{sin}}\left( {\omega t - \varphi _{{\mathrm{CEP}}}} \right)$. This is in contrast to the fact that a net current driven by a light-field is zero in the linear regime. Such a non-linear current can break the symmetry of the charge density in momentum space (or equivalently a breaking of the spatial inversion symmetry in the sense that the induced current is no longer described as an odd function of *E*(*t*) of the moment), resulting in current-induced second harmonic generation (SHG)^13–16^. Thus, the SHG (and the spatial inversion symmetry breaking) is induced even in the centrosymmetric system, if a non-linear current is driven by light.

Our target material is a layered organic superconductor κ-(BEDT–TTF)_2_Cu[N(CN)_2_]Br^17–21^ with a transition temperature *T*_SC_ = 11.6 K (Fig. 1c). Superconducting fluctuations above *T*_SC_ (*T*_SC_ < *T* < ∼2*T*_SC_) have been discussed in analogy with a pseudogap in high-*T*_SC_ superconducting cuprates in the temperature-*t*/*U*_dimer_ phase diagram^20,21^, where *t*/*U*_dimer_ is the ratio of an inter-dimer transfer integral *t* to the effective on-site Coulomb energy for a dimer *U*_dimer_, as shown in Fig. 1c. In superconductors, optical responses have been discussed in terms of non-equilibrium quasi-particle dynamics on the time scale of picosecond^22–25^, coherent excitation of the Higgs mode^26^ and light-induced superconductivity^27,28^. On the other hand, petahertz light functions, driven by the non-linear current during a light pulse is also expected to be characteristic, reflecting the ultrafast time scale of superconducting fluctuations in κ-(BEDT–TTF)_2_Cu[N(CN)_2_]Br^29^.

In this article, we report SHG for a strong light field (16 MV/cm = 1.6 V/nm) in a centrosymmetric organic superconductor κ-(BEDT–TTF)_2_Cu[N(CN)_2_]Br (single crystal). A CEP dependence of the SHG shows that it is induced by the non-linear current. Superconducting fluctuations above *T*_SC_ amplify the SHG.

## Results

### SHG and its increase near superconducting temperature

**Fig. 2:**
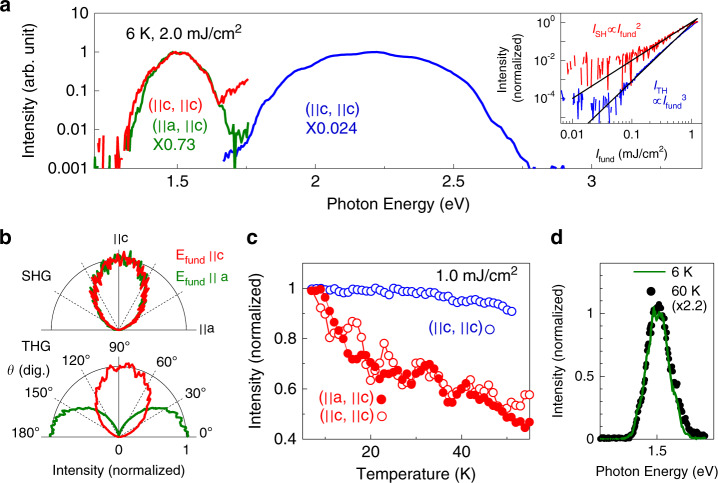
SHG and THG spectra of κ-(BEDT–TTF)2Cu[N(CN)2]Br, and their polarization and temperature dependences. **a** The red and green lines indicate the SHG spectra of single crystalline κ-(BEDT–TTF)_2_Cu[N(CN)_2_]Br (6 K) for the polarizations of (**E**_fund_ | |**c**, **E**_SH_ | | **c**) (red line) and (**E**_fund_ | |**a**, **E**_SH_ | | **c**) (green line, ×0.73). The blue line shows the THG spectrum (×0.024) for (**E**_fund_ | |**c**, **E**_TH_ | | **c**). The intensity of the fundamental light (*I*_fund_ = *E*_fund_^2^) is 2 mJ/cm^2^. Inset shows *I*_fund_ dependences of *I*_SH_ (red line for **E**_fund_ | |**c**, **E**_SH_ | | **c**) and *I*_TH_ (blue line for **E**_fund_ | |**c**, **E**_TH_ | | **c**). **b** Polarization dependences of *I*_SH_ (upper panel) and *I*_TH_ (lower panel) for **E**_fund_ | |**c** (red line) and **E**_fund_ | |**a** (green line), respectively. **c** Temperature dependences of *I*_SH_ (closed red circles: **E**_fund_ | |**a**, **E**_SH_ | | **c**, open red circles: **E**_fund_ | |**c**, **E**_SH_ | | **c**) and *I*_TH_ (open blue circles, **E**_fund_ | |**c**, **E**_TH_ | | **c**). Both are normalized by the respective intensities at 6 K. **d** SHG spectra (**E**_fund_ | |**a**, **E**_SH_ | | **c**) at 6 K (green line) and at 60 K (black dots).

Figure 2a shows spectra (measured at 6 K) of SHG and third harmonic generation (THG) with peak energies of 1.5 eV [SHG, red (**E**_Fund_ | |**c**, **E**_SH_ | | **c**) and green (**E**_Fund_ | |**a**, **E**_SH_ | | **c**, ×0.73) lines] and 2.2 eV [THG, blue line (**E**_Fund_ | |**c**, **E**_SH_ | | **c**, ×0.024)] for the fundamental photon energy of 0.75 eV. Here, **E**_Fund_, **E**_SH_, and **E**_TH_ indicate the electric fields of the fundamental light, SHG and THG, respectively. Note that the SHG is originally forbidden in this centrosymmetric system (orthorhombic with *P*nma symmetry^30,31^) in a conventional perturbation theory. However, the SHG is observed, and it is ca. 1/50 times as intensive as the THG.

As shown in the upper panel of Fig. 2b, the SHG is polarized parallel to the **c**-axis (**E**_SH_ | | **c**) for both excitation polarizations [**E**_Fund_ | |**c** (red line), **E**_Fund_ | |**a** (green line)], although the THG shows the usual polarization which is the same as that of the fundamental pulse (lower panel). Such unusual polarization dependence of the SHG is discussed below. We performed SHG and THG measurements with an incident angle of smaller than three-degree to minimize surface SHG^32^. In fact, the results above (**E**_Fund_ | |**c**, **E**_SH_ | | **c**) and (**E**_Fund_ | |**a**, **E**_SH_ | | **c**) are confirmed by rotating the sample by 90° not to depend on the *s*-polarized or *p*-polarized configuration under such a restricted condition, although the *s*-polarized or *p*-polarized configuration is indistinguishable for the incident angle of 0°. Moreover, the dependences on the CEP and the temperature (which are shown below) are incompatible with SHG induced by static geometries such as a surface or a point defect. Thus, the observed SHG is not attributed to surface SHG. The possibility of symmetry breaking induced by photo-carrier generation near the surface is also excluded by the fact that the lifetime of carriers (ca. 500 fs)^29^ (Supplementary note 3) is much longer than the time scale of the non-linear effect which drives the SHG (ca. 40 fs is evaluated from the bandwidth as discussed later).

Figure 2c shows the temperature dependence of the peak intensities for SHG [*I*_SH_: closed red circles (**E**_fund_ | |**a**, **E**_SH_ | | **c**), open red circles (**E**_fund_ | |**c**, **E**_SH_ | | **c**)] and THG (*I*_TH_: blue) (normalized by the intensities at 6 K), indicating that the SHG grows up toward *T*_SC_ from high temperature, although *I*_TH_ does not depend on the temperature. The fluctuation of *I*_SH_ for (**E**_fund_ | |**c**, **E**_SH_ | | **c**) is larger than that for (**E**_fund_ | |**a**, **E**_SH_ | | **c**) because of stray lights from the intense/ broad THG. The spectral shape is almost independent of the temperature in the range between 6 and 60 K as shown in Fig. 2d, showing that the net intensity of the SHG is increased near *T*_SC_. Such temperature dependence of the SHG, which cannot be understood by a conventional perturbation theory, clearly shows that the observed SHG is related to the superconducting fluctuations^20,21^.

### CEP dependence of SHG

Considering that the pulse width of 6 fs is shorter than the lower limit of the electron–electron scattering time [ca. 40 fs = *h*/(inter-dimer transfer integral)] where the transfer integral is about 0.1 eV in organic super conductors, a possible mechanism for the unconventional SHG is the non-linear current discussed above. Furthermore, the temperature dependence (Fig. 2c) shows that the non-linear current is enhanced by the superconducting fluctuations.

**Fig. 3:**
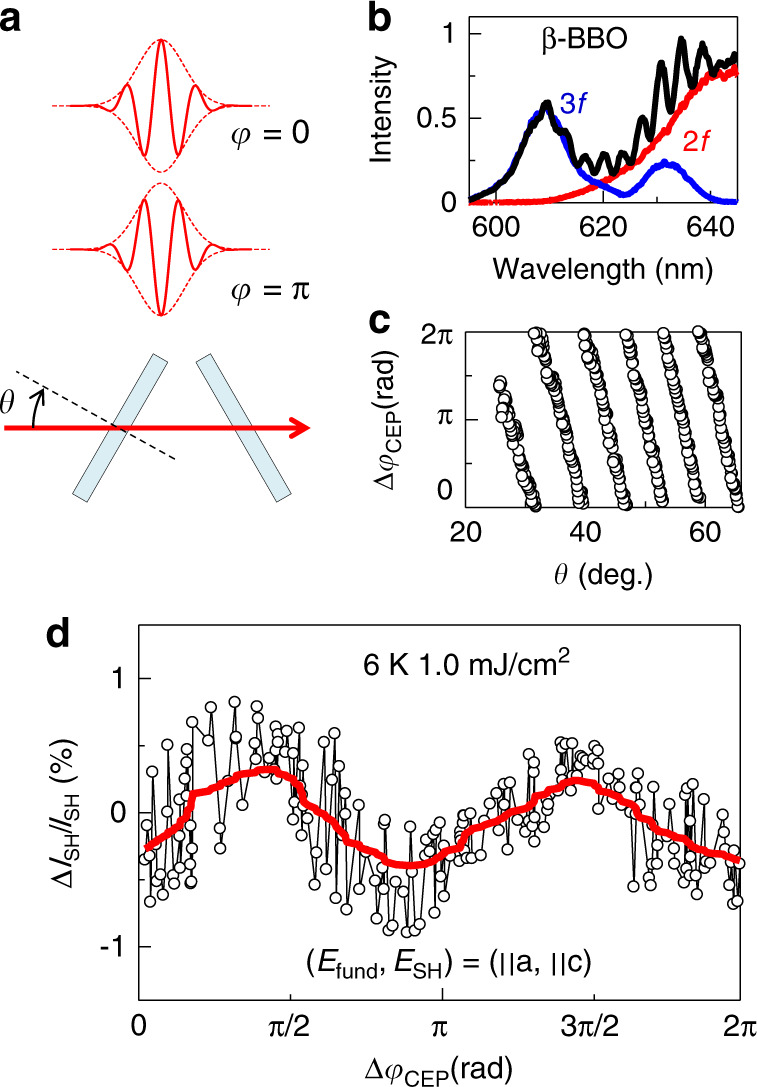
CEP control of SHG. **a** Schematic illustrations of the CEP (*φ*_CEP_ = 0, *π*) and the pair of BK-7 plates (thickness 1 mm) for changing the relative CEP Δ*φ*_CEP_ (*θ*: incident angle of light). **b** Interference spectrum between 2*f* and 3*f* of the fundamental light. The 2*f* and 3*f* are generated using β-BBO. **c** Δ*φ* as a function of *θ* (which is obtained by the 2*f*–3*f* interferometer). **d** Intensity change of the SHG (Δ*I*_SH_/*I*_SH_) as a function of Δ*φ*. The red curve is obtained by averaging the data (guide to the eye).

We can demonstrate relevance of this scenario by measuring the CEP dependence of the SHG, because the non-linear current is sensitive to the CEP as mentioned above (Fig. 1b). Figure 3d shows the intensity of the SHG as a function of the relative CEP (Δ*φ*_CEP_). During a period of the CEP, the SHG shows two maxima at around Δ*φ*_CEP_ = 1/2*π* and 3/2*π*. Considering that the directions of the light-induced current cannot be distinguished by the SHG measurement, i.e., that currents with a same amplitude and opposite directions give same SHG intensities, the above Δ*φ*_CEP_ dependence is quite reasonable. This CEP dependence of the SHG is completely consistent with the fact that the unconventional SHG is attributed to the non-linear light-induced current.

## Discussion

**Fig. 4:**
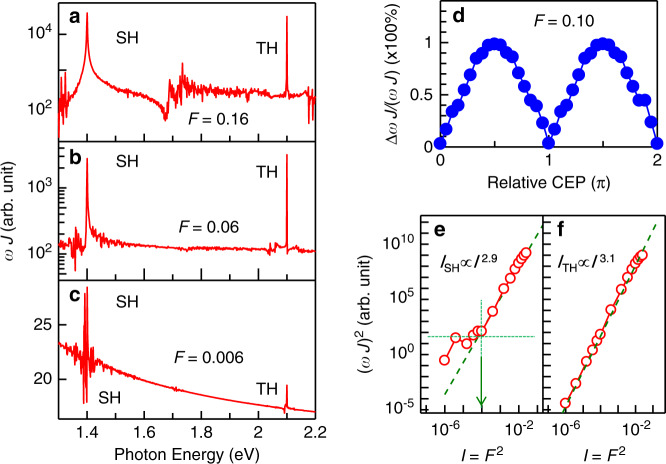
Calculated spectra of SHG and THG. **a**–**c** Calculated spectra of *ωJ* showing SHG and THG for light-field (which is polarized parallel to the **c-**axis) *F* = 0.16 **a**, 0.06 **b**, and 0.006 **c**, respectively. **d** CEP dependence of the calculated SHG intensity [peak intensities of *ωJ* at 1.4 eV (SHG)] for *F* = 0.1. **e**, **f** Calculated intensities of SHG **e** and THG **f** as a function of *I* = *F*^2^. The green dashed lines in **e** and **f** indicate *I*^2.9^ **e** and *I*^3.1^ **f**.

To clarify the origin of the SHG more in detail, we theoretically calculate the current density **j** in a two-dimensional three-quarter-filled Hubbard model for a 98 × 98-site system in the framework of the time-dependent Hartree–Fock approximation. The details are described in Supplementary note 1, where the emergence of SHG is also checked by the exact diagonalization method for a 16-site system. The calculated SHG and THG spectra (*ωJ*, *J* denotes the absolute value of the Fourier transform of **j**^8,9^) for **E**_SH_ | | **c**, **E**_TH_ | | **c** with electric field amplitudes (*F* [V/Å]) of 0.16, 0.06, and 0.006 ($\hbar \omega$ = 0.7 eV) are, respectively, shown in Fig. 4a–c. We calculate ${\mathbf{j}}\left( t \right)$ for 500 cycles of the light field. The calculated bandwidth is basically determined by the time window of the Fourier transform in the Hartree–Fock simulation without dephasing. Therefore, we cannot discuss the calculated bandwidth. *I*_SH_ is sensitive to the CEP (Fig. 4d), which is consistent with the result shown in Fig. 3d. On the other hand, the observed anisotropy of the SHG (Fig. 2b) cannot be reproduced by the theory, i.e, *I*_SH_ (theory) shows the polarization that is parallel to the fundamental polarization for both **E**_fund_ | |**c** and **E**_fund_ | |**a**. The polarization dependence cannot be understood simply by a point group analysis of *χ*^(2)^ tensor for the orthorhombic structure [class mm2(C_2v_) (after due consideration of the symmetry breaking uniaxially induced by **j**)] (Supplementary note 2). However, the results of transient reflectivity measurements do not contradict the above results of the SHG, i.e., the responses of **E**_pr_ | |**c** are larger than **E**_pr_ | |**a** for both **E**_pu_ | |**a** and **E**_pu_ | |**c** (**E**_pu_ and **E**_pr_ are the electric fields of pump-lights and probe-lights, respectively) (Supplementary note 3). This result shows that **E**_fund_ | |**a** induces charge motion along the **c**-axis.

Resistivity anisotropy under hole doping is attributed to the van Hove singularity in the density of states^33^. Under photoirradiation, the situation is similar in the sense that the carriers in the HOMO band is largely transferred into an otherwise empty band through intramolecular charge motion by the strong light field. It is noteworthy that the van Hove singularity, which is located on the *Z* point, corresponds to the momentum along the **c**-axis. Because the remaining carriers in the HOMO band would possess momenta mainly along the **c**-axis, it is natural for charge to oscillate along the **c**-axis. Considering such anisotropic cooperativity, the polarization dependence is attributed to the van Hove singularity.

As to the calculated excitation intensity (*I*, defined as $F^2$) dependences [Fig. 4e (SHG) and Fig. 4f (THG) for electrons in the HOMOs], we notice that $I_{{\mathrm{SH}}} \propto I^{2.9}$ for *I* > 10^−4^ and $I_{{\mathrm{TH}}} \propto I^{3.1}$ for *I* > 10^−6^ as shown by the green dashed lines. Above the threshold in the theory (Supplementary note 4), $I_{{\mathrm{SH}}} \propto I^{2.9}$ does not agree with the experimental result $I_{{\mathrm{SH}}} \propto I^{2.1}$ (inset of Fig. 2a). It would be caused by the fact that the intra-molecular charge motion is not taken into account, though the intra-dimer charge motion is fully taken into account. In fact, the Fourier intensity of the time profile of the charge density in the HOMO of a molecule (to which the intra-molecular optical transition is sensitive) shows a square dependence, which is consistent with our experimental result (Supplementary note 5).

Another important issue is that the spectral bandwidth of the SHG (130 meV) in Fig. 2a is narrower than that of the 6-fs excitation pulse [almost equal to the Fourier limit of the pulse (500 meV)]. The 0.6–2 eV is known as a spectral window in the organic superconductors, i.e., we have no clear reflectivity and absorption bands for | |**c** polarization. Therefore, a spectral deformation of the SHG (**E**_SH_ | | **c**) owing to an absorption loss is not the reason of the narrow bandwidth (Supplementary note 6). Considering that the band width of the THG (430 meV) is close to that of the 6-fs excitation pulse, the coherence of the SHG survives ca. 30 fs after the light-field application. Note that the wavelength dispersion of the phase-matching condition of SHG for the reflection configuration is as small as that of THG. The narrow bandwidth of the SHG is not attributed to that.

The coherence time of the SHG (30 fs) is comparable to the electronic scattering time of ∼40 fs (=*h*/(0.1 eV)) in organic superconductors, indicating that the coherence time is governed by the electronic scattering (Supplementary note 7). This is consistent with the fact that the bandwidth of the SHG is independent of temperature as shown in Fig. 2d. On the other hand, the intensity increases toward *T*_SC_, suggesting that such non-linear charge motion on the length scale of a few molecules is enhanced by the superconducting fluctuations. Thus, 6-fs field application on the time scale much shorter than the electron scattering time of ca. 40 fs is an issue very different from the HHG driven by terahertz and mid-infrared fields, where the electric field continues to be applied even after a substantial number of scatterings occur. This is a very important perspective for petahertz functions and attosecond science of superconductors and of strongly correlated systems in addition to already realized terahertz functions of them^15,16,26,27^.

In summary, this article demonstrates SHG in the centrosymmetric organic superconductor κ-(BEDT–TTF)_2_Cu[N(CN)_2_]Br. The SHG is enhanced toward *T*_SC_. A narrow bandwidth of 130 meV shows that the coherence of the SHG survives for 30 fs after the 6 fs light-field application. The CEP dependence indicates that the SHG is induced by the non-linear petahertz current.

## Methods

### Sample preparation

Single crystals of κ-(BEDT–TTF)_2_Cu[N(CN)_2_]Br were grown under dried nitrogen gas atmosphere by electrochemical oxidation of BEDT–TTF (80 mg) dissolved in 100 ml of 1, 1, 2-trichloroethane (10% v/v ethanol) in the presence of supporting electrolytes NaN(CN)_2_, CuBr, and 18-crown-6 ether^29^. A thick plate-like crystal (0.7 × 0.5 × 0.8 mm for **a**, **b**, and **c**-axes, respectively) was selected for optical measurements. The **a*–*c** crystal plane corresponds to the two-dimensional conductive layer.

### 6 fs infrared pulse generation

A broadband infrared spectrum covering 1.2–2.3 μm of the 6 fs pulse is obtained by focusing a carrier-envelope phase (CEP) stabilized idler pulse (1.7 μm) from an optical parametric amplifier (Quantronix HE-TOPAS pumped by Spectra-Physics Spitfire-Ace) onto a hollow fiber set within a Kr-filled chamber (Femtolasers). Pulse compression is performed using both active mirror (OKO, 19-ch linear MMDM) and chirped mirror (Femtolasers and Sigma-Koki) techniques^29,34^.

### Measurement and CEP control of SHG

We performed SHG and THG measurements for the single crystal using a 6 fs pulse with a reflection geometry (incident angle is smaller than 3°). The intensity and polarization of the fundamental (excitation range: 0.01–2 mJ/cm^2^) pulse are controlled by a pair of wire-grid CaF_2_ polarizers^34^. The SHG and the THG are detected by a photomultiplier tube (Hamamatsu R13456) after passing through a spectrometer (JASCO, M10). The CEP of the fundamental pulse is controlled by a pair of glass plates with the incidence angle of *θ* (Fig. 3a) and detected by the 2*f*–3*f* interferometer (2*f* and 3*f* are generated using β-BBO) (Fig. 3b, c).

## Supplementary information

Supplementary Information

## Data Availability

The data that support the plots within this paper and other findings of this study are available from the corresponding author upon reasonable request.

## Code availability

All relevant codes are available from the corresponding author upon reasonable request.
