# Supplementary material for: Petahertz non-linear current in a centrosymmetric organic superconductor
Source: Nat Commun. 2020 Aug 18;11:4138. doi: 10.1038/s41467-020-17776-3 (PMC7434879; doi:10.1038/s41467-020-17776-3)
Supplement: Supplementary file 1 — Supplementary Information [file 41467_2020_17776_MOESM1_ESM.pdf]

Supplementary information for

Petahertz non-linear current in a centrosymmetric organic superconductor

Y. Kawakami[1], T. Amano[1], H. Ohashi[1], H. Itoh[1], Y. Nakamura[2], H. Kishida[2], T. Sasaki[3], G. Kawaguchi[4], H. M. Yamamoto[4], K. Yamamoto[5], S. Ishihara[1], K. Yonemitsu[6], and S. Iwai[1*]

[1]*Department of Physics, Tohoku University, Sendai 980-8578, Japan*
[2]*Department of Applied Physics, Nagoya University, Nagoya 464-8603, Japan*
[3]*Institute for Materials Research, Tohoku University, Sendai 980-8577, Japan*
[4]*Institute for Molecular Science, Okazaki 444-8585, Japan*
[5]*Department of Applied Physics, Okayama University of Science, Okayama, 700-0005, Japan*
[6]*Department of Physics, Chuo University, Tokyo 112-8551, Japan*

71.27.+a, 74.25.Gz, 78.47.J-

* s-iwai@tohoku.ac.jp

Supplementary note 1

Technical details of theoretical consideration (2D Hubbard model)

We use the Hubbard model at three-quarter filling,

$H = \sum_{\langle i,j\rangle\sigma} t_{ij}\left(c_{i\sigma}^{\dagger}c_{j\sigma} + c_{j\sigma}^{\dagger}c_{i\sigma}\right) + U\sum_i n_{i\uparrow}n_{i\downarrow}$, where $c_{i\sigma}^{\dagger}$ creates an electron in the highest occupied molecular orbital (HOMO) with spin σ at site i, and $n_{i\sigma} = c_{i\sigma}^{\dagger}c_{i\sigma}$. The parameter U represents the on-site Coulomb repulsion and U=0.8 eV is used. The transfer integral t_{ij} depends on the bond ij. The molecular arrangement is taken from the structural data, from which t_{ij} are estimated with the extended Hückel method [1, 2]. The initial state is the Hartree-Fock ground state. Photoexcitation is introduced through the substitution $c_{i\sigma}^{\dagger}c_{j\sigma} \rightarrow \exp\left[\frac{ie}{\hbar c}\mathbf{r}_{\mathrm{ij}}\cdot\mathbf{A}(t)\right]c_{i\sigma}^{\dagger}c_{j\sigma}$ with relative intermolecular

position $\mathbf{r}_{ij} = \mathbf{r}_j - \mathbf{r}_i$. We use the vector potential $\mathbf{A}(t) = \theta(t)\frac{\mathbf{F}}{\omega_{\text{fund}}}[\cos(\omega_{\text{fund}}t - \varphi) - \cos\varphi]$, which corresponds to $\mathbf{E}(t) = \theta(t)\,\mathbf{F}\sin(\omega_{\text{fund}}t - \varphi)$, with $\mathbf{F} = (0, F)||\mathbf{c}$-axis and fundamental photon energy $\hbar\omega_{\text{fund}}$ =0.7 eV. The time-dependent Schrödinger equation is numerically solved. We calculate the Fourier transform of the current density $\mathbf{j}(t) = -\langle\frac{\partial H}{N\partial \mathbf{A}}\rangle$ with N being the number of unit cells for 500 cycles. The absolute value of its Fourier transform is denoted by J. The SHG and THG are evaluated as ωJ (the absolute value of the Fourier transform of $\mathrm{d}\mathbf{j}/\mathrm{d}t$) at ω=2ω_{fund} and ω=3ω_{fund} [3, 4], respectively. To check the emergence of SHG, we also calculate J by using the exact diagonalization method for a 16-site system during 10-cycle irradiation [5, 6]. Fourier spectra of ωJ are shown by the closed blue circles in Fig. s1 [(a) F=0.16, (b) F=0.06, (c) F=0.006]. As shown in Figs. s1(a) and s1(b), the SHG peak is confirmed.

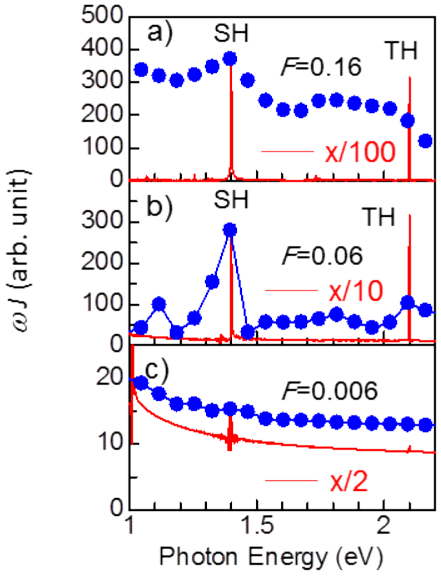

Figure s1 Calculated spectra of SHG (exact diagonalization)

a, **b**, **c** Calculated spectra of ωJ showing SHG and THG by the closed blue circles ($\mathbf{E}_{\text{fund}}$ $||$**c**, **j**$||$**c**) for F= 0.16(**a**), 0.06 (**b**), and 0.006 (**c**), respectively. The spectra of ωJ calculated by the time-dependent Hartree Fock approximation [Figs. 4(a), 4(b) and 4(c) in the main text] are shown by the red curves after normalization.

In Figs. 4(a)-4(c), the SHG is overestimated, because of the following reason. When we fully take electron correlations into account by employing the exact diagonalization method, we find that inter-site repulsive interactions substantially reduce the coherence of the charge oscillations that are responsible for the SHG, just as they reduce the coherence of the non-linear charge oscillations that are responsible for the stimulated emission [6]. On the other hand, we also find that they do not affect the THG so much. In the main text, we employ the time-dependent Hartree-Fock approximation, which cannot treat such decoherence effects, so that the SHG is overestimated and thus comparable with the THG.

Supplementary note 2
Anisotropy of SHG

The SHG is polarized to the **c**-axis ($\mathbf{E}_{\mathrm{SH}}||\mathbf{c}$) for both fundamental polarizations ($\mathbf{E}_{\mathrm{Fund}}||\mathbf{c}$, $\mathbf{E}_{\mathrm{Fund}}||\mathbf{a}$) as shown in Fig. 2(b), although the THG shows the usual polarization ($\mathbf{E}_{\mathrm{TH}}||\mathbf{c}$ for $\mathbf{E}_{\mathrm{fund}}||\mathbf{c}$ and $\mathbf{E}_{\mathrm{TH}}||\mathbf{a}$ for $\mathbf{E}_{\mathrm{fund}}||\mathbf{a}$). As described in the main text, the observed anisotropy of the SHG ($\mathbf{E}_{\mathrm{SH}}||\mathbf{c}$ for $\mathbf{E}_{\mathrm{Fund}}||\mathbf{a}$) cannot be reproduced by the theory which takes only the HOMO for each BEDT-TTF molecule into account, i. e., ($\mathbf{E}_{\mathrm{fund}}||\mathbf{c}$, $\mathbf{E}_{\mathrm{SH}}||\mathbf{c}$) and ($\mathbf{E}_{\mathrm{fund}}||\mathbf{a}$, $\mathbf{E}_{\mathrm{SH}}||\mathbf{a}$) in the calculation.

The polarization dependence of the SHG in κ-(BEDT-TTF)$_2$Cu[N(CN)$_2$]Br should be described by the $\chi^{(2)}$ tensor of the orthorhombic structure [class mm2 (C_{2v}) after due consideration of the symmetry breaking uniaxially induced by the current **j**],

$$\chi^{(2)} = \begin{pmatrix} 0 & 0 & 0 & 0 & \chi^{(2)}_{xzx} & \chi^{(2)}_{xxy} \\ 0 & 0 & 0 & \chi^{(2)}_{yyz} & 0 & 0 \\ \chi^{(2)}_{zxx} & \chi^{(2)}_{zyy} & \chi^{(2)}_{zzz} & 0 & 0 & 0 \end{pmatrix}$$

, where $\chi^{(2)}_{zxx} = \chi^{(2)}_{xzx}$, $\chi^{(2)}_{yyz} = \chi^{(2)}_{zyy}$

The relation ($\mathbf{E}_{\mathrm{fund}}||\mathbf{c}$, $\mathbf{E}_{\mathrm{SH}}||\mathbf{c}$) is easy to understand because $\chi^{(2)}_{zzz} = \chi^{(2)}_{ccc}$ is nonzero for $\mathbf{j}||\mathbf{c}$ (Uniaxial symmetry breaking is assumed in the direction of the **c**-axis). On the other hand, the observed anisotropy [($\mathbf{E}_{\mathrm{fund}}||\mathbf{a}$, $\mathbf{E}_{\mathrm{SH}}||\mathbf{c}$)

and ($\mathbf{E}_{\mathrm{fund}}$||**c**, $\mathbf{E}_{\mathrm{SH}}$||**c**)] cannot be understood by the point group analysis, since [($\mathbf{E}_{\mathrm{fund}}$||**c**, $\mathbf{E}_{\mathrm{SH}}$||**a**) and ($\mathbf{E}_{\mathrm{fund}}$||**c**, $\mathbf{E}_{\mathrm{SH}}$||**c**)] could be realized by $\chi^{(2)}{}_{zxx} = \chi^{(2)}{}_{caa}$.

Meanwhile, the results of transient reflectivity measurements are consistent with the above results, i.e., the optical responses measured by a transient reflectivity are larger for $\mathbf{E}_{\mathrm{pr}}$||**c** than for $\mathbf{E}_{\mathrm{pr}}$||**a** for both $\mathbf{E}_{\mathrm{pu}}$||**c** and $\mathbf{E}_{\mathrm{pu}}$||**a** ($\mathbf{E}_{\mathrm{pu}}$ and $\mathbf{E}_{\mathrm{pr}}$ are the electric fields of the pump- and probe- lights) [supplementary note 3]. Thus, non-linear charge motion along the **c**-axis is actually driven by $\mathbf{E}_{\mathrm{pu}}$||**a** as well.

The microscopic reason for the induction of **j**||**c** by $\mathbf{E}_{\mathrm{fund}}$ ||**a** remains unclear. However, it is reasonable to consider that the induction of **j**||**c** by $\mathbf{E}_{\mathrm{fund}}$||**a** is caused by the intra-molecular charge transfers, which are beyond the scope of the theory.

Supplementary note 3

Polarization analysis of transient reflectivity (pump-probe) measurement

To demonstrate a response of **j**||**c** ($\mathbf{E}_{pr}$||**c**) under the excitation polarization of $\mathbf{E}_{pu}$||**a**, we investigate the polarization dependence of a transient reflectivity (pump-probe) measurement. Figure s2 shows time evolutions of reflectivity changes ($\Delta R/R$) at 0.62 eV (excitation is made by 6 fs, 1 mJ/cm^2 ,

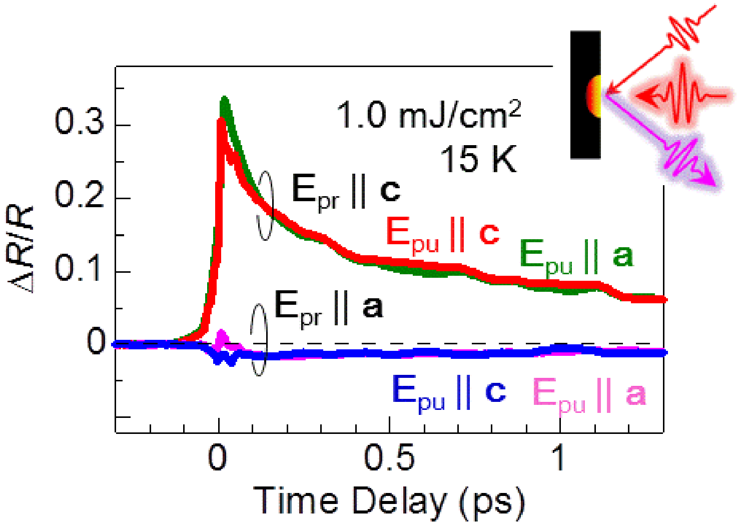

Figure s2

Polarization analysis of transient reflectivity (pump-probe) measurement

Time evolutions of transient reflectivity change [$\Delta R/R$ at 0.62 eV (excitation by 6 fs

0.6-0.9 eV). The $\Delta R/R$ for $\mathbf{E}_{pr}$||**c** [red ($\mathbf{E}_{pu}$||**c**) and green ($\mathbf{E}_{pu}$||**a**) curves] are much larger than those for $\mathbf{E}_{pr}$||**a** [blue ($\mathbf{E}_{pu}$||**c**) and magenta ($\mathbf{E}_{pu}$||**a**) curves)]. (In reference 5, only the results for $\mathbf{E}_{pu}$||**c**, $\mathbf{E}_{pr}$||**c** have been discussed). Therefore, it is clear that light-induced charge motion occurs easily along the **c**-axis for both $\mathbf{E}_{pu}$||**a** and $\mathbf{E}_{pu}$||**c**. The similar polarization dependence has also been observed in κ-(BEDT-TTF)$_2$Cu[N(CN)$_2$]Cl (insulating phase) even under weaker excitation condition of 3.1 eV (probe energy=1.55 eV)[7].

Supplementary note 4

Calculated SHG spectra below and above the threshold

We have clear difference in calculated spectra of the SHG below and above the threshold, i. e., a sharp peak at 1.4 eV [=0.7 eV (fundamental photon energy in the theory) x2] is seen above the threshold [Fig. s3(a)], whereas we notice an oscillating structure on the background of the fundamental component below the threshold [resulting in the fluctuation in Fig. 4(e)] [Fig. s3(b)]. However, the experimental threshold of the SHG is not clear because of detection limits [inset of Fig. 2(a)].

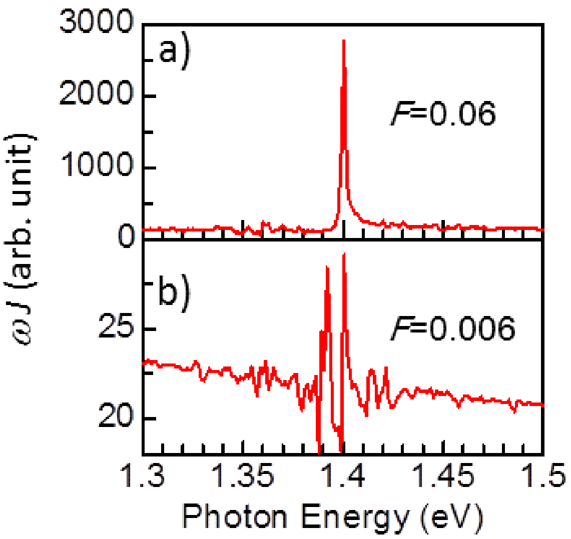

Figure s3 Calculated SHG spectra below and above the threshold

Calculated spectra of the SHG above the threshold (**a** F=0.06) and below the threshold (**b** F=0.006).

Supplementary note 5

Discrepancy between the experimental and the theory on excitation intensity dependence

$I_{SH} \propto I^{2.9}$ [Fig. 4(e)] does not agree with the experimental result $I_{SH} \propto I^{2.1}$ [inset of Fig. 2(a)]. Although the reason of this discrepancy remains unclear, it can be related to the fact that the intra-molecular charge motion is not taken into account, as mentioned in the main text. In fact, the Fourier intensity (the square of the absolute value of the Fourier transform) of the time profile of the charge density in the HOMO of a molecule to which the intra-molecular optical transition is sensitive shows square dependence above the threshold as follows. The closed blue circles in Fig. s4 show the Fourier intensities ρ^2 for the charge density during 500-cycle irradiation as for $(\omega J)^2$, and the black circles show those during 50-cycle irradiation. The red circles show the calculated SHG intensities $(\omega J)^2$ [red circles in Fig. 4(e)] . The blue, black and red dashed lines indicate $I^{2.0}$ (blue and black) and $I^{2.9}$ (red). Thus, the discrepancy between the observation ($I_{SH} \propto I^{2.1}$) and the theory ($I_{SH} \propto I^{2.9}$) would be due to the intra-molecular charge redistribution.

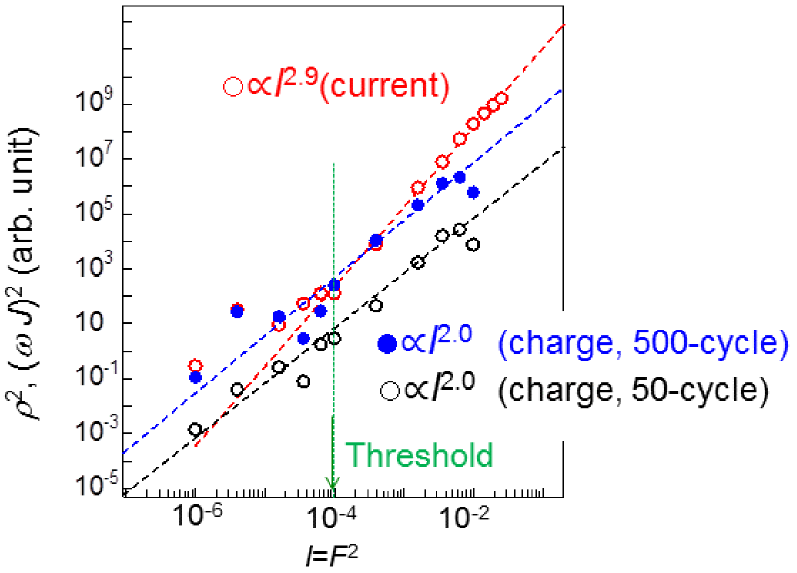

Figure s4 Charge density in the HOMO

Fourier intensities of the time profile of the charge density in the HOMO of a molecule ρ^2 as a function of $I=F^2$ (closed blue circles for those during 500-cycle irradiation and black circles for those during 50-cycle irradiation). The blue, black and red dashed lines indicate $I^{2.0}$ (blue and black) and $I^{2.9}$ (red). The red

Supplementary note 6

Transmittance spectrum of κ-(BEDT-TTF)$_2$Cu[N(CN)$_2$]Br thin crystal in near infrared - visible region.

The energy range (0.6-2 eV) is known as a spectral window between inter-molecular (< 0.5 eV) and intra-molecular (> 2.3 eV) charge transfer bands in the organic superconductors [8-10], i.e., we have no large reflectivity or absorption bands except for a peak at 1.5 eV as shown in references [11, 12]. We measure a transmittance spectrum of a thin crystal [13] to investigate the influence of the absorption to the SHG, because it is difficult to estimate an absorption coefficient by Kramers-Kronig analysis of a reflectivity in such a transparent spectral region. Thin single crystal films were grown by an electrochemical oxidation method in a similar manner to that for the bulk crystals. The detail of thin crystal growth was described in

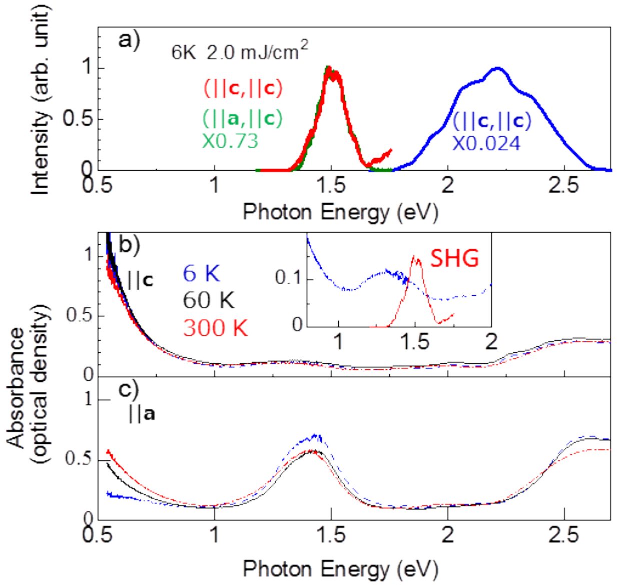

Figure s5

Transmittance spectrum of κ-(BEDT-TTF)$_2$Cu[N(CN)$_2$]Br thin cryctal

(a) SHG (red and green curves) and THG (blue curve) spectra (same as Fig. 2(a), but shown by a linear scale). **(b)(c)** absorption spectra of a κ-(BEDT-TTF)$_2$Cu[N(CN)$_2$]Br thin crystal with a thickness of 150 nm [13] for **E**||**c** (b) and **E**||**a**(c) (6K; blue, 60 K; black, 300 K; red). ***E*** is the electric field of light. Inset in (b) shows an enlarged view of the intramolecular transition (***E***||**c**) and the SHG.

[13]. A thin crystal with a thickness of 150 nm was picked up and transferred to the top of a CaF_2 substrate.

Figures s5(b)(c) show absorption spectra of the κ-(BEDT-TTF)$_2$Cu[N(CN)$_2$]Br thin crystal film [13] for **E**||**c** (b) and **E**||**a** (c) (**E** is the electric field of light) [Fig. s5(a) shows the SHG and THG (linear scale)]. The peak structure at 1.37 eV for **E**||**a** is attributed to the intra-molecular transition of a BEDT-TTF molecule. The anisotropy of this intra-molecular transition, i. e. the peak for **E**||**a** is much larger than that for **E**||**c**, is associated with the crystal structure of κ-(BEDT-TTF)$_2$Cu[N(CN)$_2$]Br, where the long axis of BEDT-TTF molecules is tilted towards the **a**-axis [1, 2, 14]. The small and broad spectral feature around 1.5 eV for **E**||**c** [inset of Fig. s5(b)] does not affect the spectral shapes of the SHG for both ($\mathbf{E}_{\mathrm{fund}}$||**c**, $\mathbf{E}_{\mathrm{SH}}$||**c**) and ($\mathbf{E}_{\mathrm{fund}}$||**a**, $\mathbf{E}_{\mathrm{SH}}$||**c**) [Fig. 2(a)]. Thus, a spectral deformation of the SHG ($\mathbf{E}_{\mathrm{SH}}$||**c**) owing to an absorption loss is not the reason of the narrow bandwidth.

Supplementary note 7

Coherence time of non-linear charge motion as studied by transient reflectivity measurement with double pump pulses

The electronic coherence after the field application is also seen in another strong light field effect in this compound [5]. We perform a transient reflectivity ($\Delta R/R$) measurement with a double-pump pulse to make an insight of coherence induced by a strong-light field. This method has been employed for elucidating electronic and vibrational coherence [15-17]. Here, the coherence of the non-linear charge motion, which has been assigned as the origin of stimulated emission[5], is discussed.

Figures s6(a)(b) show an interferogram pattern which is detected by a $\Delta R/R$ measurement with double pump pulses [as a function of Δt [=time difference between two pump pluses; -20-110 fs (a), -2-17 fs (b)]. It is probed at t_d [=time delay between the 1st pump and the probe (0.62 eV= peak of stimulated emission) pulses]= 50 fs. An interferogram reflecting the coherence of the non-linear charge motion [5] [red curves in Figs. s6 (a) and s6(b)] indicates that electronic coherence survives ca. 70 fs after the pulse [A black curve in (b) shows the autocorrelation of the pulse]. The Fourier spectrum of the interferogram [red curve in Fig. s6(c)] is analogous to the spectrum of the stimulated emission reflecting the non-linear charge motion observed in the

single-pumped measurement [5] [Fig. s6(d)].

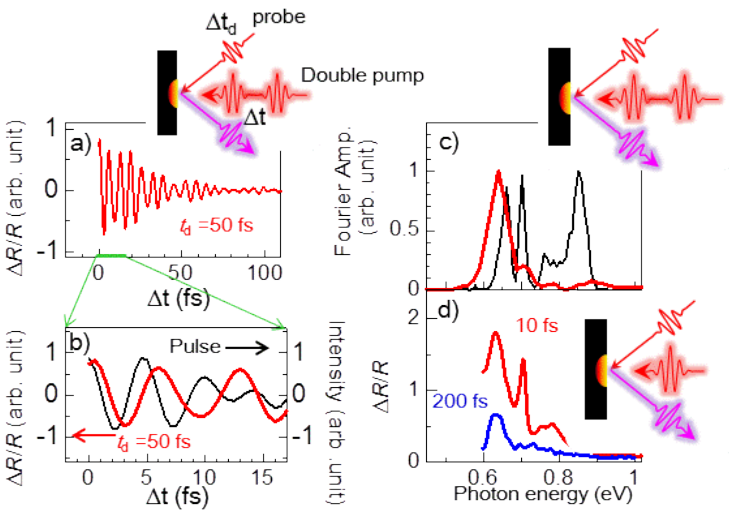

Figure s6 Coherence time of the non-linear charge oscillation

a, b Interferogram pattern which is detected by a transient reflectivity (Δ*R/R*) measurement with double pump pulses [as a function of Δ*t* (=time difference between two pump pluses) covering the range of -20-110 fs (**a**), -2-17 fs (b)]. It is probed at t_d [=time delay between the 1st pump and the probe (0.62 eV= peak of stimulated emission[5]) pulses]= 50 fs. The black line in **b** shows autocorrelation of the 6 fs pump pulse. **c** Fourier spectra of the interferogram (red curve) and the autocorrelation of the pulse (black curve). **d** Spectra of stimulated emission which are measured by the single-pump transient reflectivity measurement (t_d=10 fs(red curve), 200 fs(blue curve))[5].

This result confirms that the spectral band width of Δ*R/R* in the single-pumped measurement is determined by the coherence time of the non-linear charge motion. Thus, the electronic coherence of the non-linear charge motion can survive after the pulse. The coherence time of the stimulated emission (ca. 70 fs) and the SHG (30 fs) are comparable to the electronic scattering time of ~40 fs (=h/(0.1 eV)) in organic conductors.

Reference (Supplementary notes)

[1] Mori, T., Mori, H. & Tanaka, S. Structural genealogy of BEDT-TTF-based organic conductors II. Inclined molecules: θ, α, and κ phases. Bull. *Chem. Soc. Jpn.* **72**, 179-197 (1999).

[2] Watanabe, M. et al. Low temperature superstructure and transfer integrals in κ-(BEDT-TTF)$_2$Cu[N(CN)$_2$]X: X = Cl, Br. *Synth. Met.* **103**, 1909-1910 (1999).

[3] Silva, R. E. F. et al. High-harmonic spectroscopy of ultrafast manybody dynamics in strongly correlated systems. *Nat. Photon.* **12**, 266-270(2018).

[4] Murakami, Y., Eckstein, M. & Werner, P. High-harmonic generation in Mott insulators. *Phys. Rev. Lett.* **121**, 057405(2018).

[5] Kawakami, Y. et al. Nonlinear charge oscillation driven by a single-cycle light field in an organic superconductor. *Nat. Photon.* **12**, 474(2018).

[6] Yonemitsu, K. Photoinduced high-frequency charge oscillations in dimerized systems. *J. Phys. Soc. Jpn.* **87**, 044708 (2018).

[7] Tsuchiya, S., Nakagawa, K., Taniguchi, H. & Toda, Y. Polarization-resolved ultrafast spectroscopy in an organic Mott insulator κ-(BEDT-TTF)$_2$Cu[N(CN)$_2$]Cl. *J. Phys. Soc. Jpn.* **88**, 074706 (2019).

[8] Sasaki, T. et al. Electronic correlation in the infrared optical properties of the quasi-two-dimensional κ-type BEDT-TTF dimer system. *Phys. Rev. B* **69**, 064508 (2004).

[9] Dressel, M. & Drichko, N. Optical properties of two-dimensional organic conductors: Signatures of charge ordering and correlation effects. *Chem. Rev.* **104**, 5689-5715 (2004).

[10] Faltermeier, D. et al. Bandwidth-controlled Mott transition in κ-(BEDT-TTF)$_2$Cu[N(CN)$_2$]Br$_x$Cl$_{1-x}$: optical studies of localized charge excitations. *Phys. Rev.* **B76**, 165113 (2007).

[11] Tamura, M. et al. Reflectance spectra of κ-(BEDT-TTF)$_2$I$_3$: Electronic structure of dimeric BEDT-TTF salts. *J. Phys. Soc. Jpn.* **60**, 3861-3873(1991).

[12] Nakamura, Y. et al. Optical properties of a quantum spin liquid candidate material. κ-(BEDT-TTF)$_2$Ag$_2$(CN)$_3$. *J. Phys. Soc. Jpn.* **86**, 014710 (2017).

[13] Yamamoto, H. M. et al. A strained organic field-effect transistor with a gate-tunable superconducting channel. *Nat. Commun.* **4**, 2379 (2013).

[14] Sedlmeier, K. et al. Absence of charge order in the dimerized κ- phase BEDT-TTF salts. *Phys. Rev. B* **86**, 245103(2012).

[15] Yusupov, R. et al. Coherent dynamics of macroscopic electric order through a symmetry breaking transition. *Nat. Phys.* **6**, 681(2010).

[16] Matsubara, Y. et al. Coherent dynamics of photoinduced phase formation in a strongly correlated organic crystal, *Phys. Rev. B* **89**, 161102(R)(2014).

[17] Sasaki, H. et al. Coherent control theory and experiment of optical phonons in diamond, *Sci. Rep.* **8**, 9609(2018).
